# Endocytosis of Multiwalled Carbon Nanotubes in Bronchial Epithelial and Mesothelial Cells

**DOI:** 10.1155/2015/793186

**Published:** 2015-05-18

**Authors:** Kayo Maruyama, Hisao Haniu, Naoto Saito, Yoshikazu Matsuda, Tamotsu Tsukahara, Shinsuke Kobayashi, Manabu Tanaka, Kaoru Aoki, Seiji Takanashi, Masanori Okamoto, Hiroyuki Kato

**Affiliations:** ^1^Institute for Biomedical Sciences, Interdisciplinary Cluster for Cutting Edge Research, Shinshu University, 3-1-1 Asahi, Matsumoto, Nagano 390-8621, Japan; ^2^Department of Orthopaedic Surgery, Shinshu University School of Medicine, 3-1-1 Asahi, Matsumoto, Nagano 390-8621, Japan; ^3^Clinical Pharmacology Educational Center, Nihon Pharmaceutical University, 10281 Komuro, Ina-machi, Saitama 362-0806, Japan; ^4^Department of Molecular Pharmacology and Neuroscience, Nagasaki University Graduate School of Biomedical Sciences, 1-14 Bunkyo-machi, Nagasaki 852-8521, Japan

## Abstract

Bronchial epithelial cells and mesothelial cells are crucial targets for the safety assessment of inhalation of carbon nanotubes (CNTs), which resemble asbestos particles in shape. Intrinsic properties of multiwalled CNTs (MWCNTs) are known to cause potentially hazardous effects on intracellular and extracellular pathways. These interactions alter cellular signaling and affect major cell functions, resulting in cell death, lysosome injury, reactive oxygen species production, apoptosis, and cytokine release. Furthermore, CNTs are emerging as a novel class of autophagy inducers. Thus, in this study, we focused on the mechanisms of MWCNT uptake into the human bronchial epithelial cells (HBECs) and human mesothelial cells (HMCs). We verified that MWCNTs are actively internalized into HBECs and HMCs and were accumulated in the lysosomes of the cells after 24-hour treatment. Next, we determined which endocytosis pathways (clathrin-mediated, caveolae-mediated, and macropinocytosis) were associated with MWCNT internalization by using corresponding endocytosis inhibitors, in two nonphagocytic cell lines derived from bronchial epithelial cells and mesothelioma cells. Clathrin-mediated endocytosis inhibitors significantly suppressed MWCNT uptake, whereas caveolae-mediated endocytosis and macropinocytosis were also found to be involved in MWCNT uptake. Thus, MWCNTs were positively taken up by nonphagocytic cells, and their cytotoxicity was closely related to these three endocytosis pathways.

## 1. Introduction

Carbon nanotubes (CNTs) were first discovered by Oberlin et al. [1], and they have attracted increasing attention since the end of 20th century. Owing to their unique physical, mechanical, and electronic properties, CNTs serve as valuable reinforcements or enhance the properties and introduce novel functionalities of various materials in a number of fields, including chemistry, electronics, energy, and materials science [2, 3]. The unique properties of CNTs have also garnered considerable attention from the fields of medicine and biology, and they have potential applications as biomaterials for biosensors, drug and vaccine delivery vehicles, and scaffold materials [4–6].

However, the potential adverse effects of CNTs on human health are of great concern, considering their increasing use in composite biomaterials and exploration as innovative solutions for biomedical applications or in nanomedicine as well as the potential workplace exposure [7–9]. CNTs possess asbestos-like morphological characteristics (i.e., a nanoscale size and a high aspect ratio) and persist in the human body for a long time [10–12]. In 2008, Takagi et al. reported that transgenic mice intraperitoneally injected with MWCNTs exhibited mesothelioma similar to that in mice exposed to asbestos [13]. Subsequently, induction of mesothelioma was also reported after intraperitoneal or intrascrotal injection of CNTs in rodents [14–16]. Moreover, some evidence suggests that CNT causes cancer upon inhalation or intratracheal administration [17–20], although there is no direct evidence that CNTs induce pleural mesothelioma and lung cancer [17, 21–24].

Previous studies have clarified the carcinogenic mechanisms of CNTs in vitro. The number of micronuclei in lung epithelial cells increases upon exposure to MWCNTs, which is indicative of genotoxicity such as chromosomal damage or mitotic spindle disruption [20]. Sargent et al. showed that CNTs induce mitotic spindle disruption that results in errors in chromosome number [8, 25–27]. CNTs must be internalized by cells for such phenomena to occur. We have previously reported that it is important for multiwalled CNTs (MWCNTs) to be internalized for cytotoxic effects to be observed in a human mesothelioma cell line (MESO-1) and a human bronchial epithelial cell line (BEAS-2B) [28–30]. However, the internalization mechanism of CNTs is not well known.

In this study, we demonstrated the mechanism underlying CNT internalization in human primary bronchial epithelial cells and mesothelium cells. Moreover, we also demonstrated the internalization mechanism of CNTs in nonphagocytic cells by using various endocytosis inhibitors.

## 2. Materials and Methods

### 2.1. Carbon Nanotubes

MWCNTs manufactured by a chemical vapor deposition method [31] were provided by Hodogaya Chemical (MWNT-7; Tokyo, Japan); their properties have been reported previously [32]. The sterilization conditions were autoclaving at 121°C for 15 min. MWCNTs were vortexed for 1 min in 0.1% gelatin (MediGelatin; Nippi, Tokyo, Japan) or 2% fetal bovine serum (FBS; Life Technologies, Grand Island, NY, USA) in phosphate-buffered saline (PBS) and sonicated for 30 min. MWNT-7 was diluted if required, and a volume of 1/100 was added to the cell culture fluid in the following exposure experiments.

### 2.2. Endocytosis Inhibitors

The endocytosis inhibitors used were previously described by Yumoto et al. [33]. Phenylarsine oxide, indomethacin, nystatin, and 5-(N-ethyl-N-isopropyl)amiloride were purchased from Sigma-Aldrich (St. Louis, MO, USA). Chlorpromazine was purchased from Nacalai Tesque (Kyoto, Japan). Phenylarsine oxide was dissolved in dimethyl sulfoxide (DMSO) and diluted to 0.2–5 mM. Indomethacin was dissolved in ethanol at 50°C and diluted to 5–100 mM. Nystatin was dissolved in DMSO and diluted to 1–20 mM. 5-(N-Ethyl-N-isopropyl)amiloride was dissolved in DMSO and diluted to 5–80 mM. Chlorpromazine was dissolved in PBS and diluted to 2–50 mM.

### 2.3. Cell Culture

Normal human bronchial epithelial cells (HBECs) were purchased from Lonza (Walkersville, MD, USA). Normal human mesothelial cells (HMCs) were purchased from Zen-Bio, Inc. (Research Triangle Park, NC, USA). The BEAS-2B human bronchial epithelial cell line was purchased from American Type Culture Collection (Manassas, VA, USA). The ACC-MESO-1 human malignant pleural mesothelioma cell line [34] was purchased from RIKEN (Ibaraki, Japan). HBECs were cultured in bronchial/tracheal epithelial cell serum-free growth medium kit with 0.1 *μ*g/mL retinoic acid (Cell Application, San Diego, CA, USA) and passaged every 4 d, with the medium exchanged every alternate day. HMCs were cultured in mesothelial cell growth medium (Zen-Bio, Inc.) and passaged twice a week. Both types of normal cell were used within 5 passages. BEAS-2B cells were cultured in Ham's nutrient mixture F-12 (Nacalai Tesque) with 10% FBS and passaged twice a week. MESO-1 cells were cultured in RPMI 1640 (Nacalai Tesque) with 10% FBS and passaged twice a week. For each experiment, the cells were seeded at a density of 2 × 10^5^ cells/cm^2^ and allowed to adhere for 24 h.

### 2.4. Cell Viability

The cell viability assay was performed as described previously [35]. We performed an Alamar Blue assay (alamarBlue cell viability reagent; Invitrogen, Carlsbad, CA) according to the manufacturer's instructions. Cells were plated in 96-well plates and incubated for 24 h at 37°C in the culture medium containing MWCNTs in a dispersant or in a control medium containing only dispersant without MWCNTs. Viable cells metabolized the dye, resulting in increased fluorescence detected by excitation/emission at 530/590 nm using a fluorescence multiplate reader (PowerScan 4; DS Pharma Biomedical, Osaka, Japan). Cell viability was calculated as follows: percent cytotoxicity = 100 × experimental value/control value. The test media were assayed six times for each treatment condition.

### 2.5. Imaging of MWNT-7 Uptake by Fluorescence Microscopy

Cells were cultured on ibiTreat *μ*-Slide (ibidi GmbH, Martinsried, Germany) for snapshot imaging and ibiTreat *μ*-dish for time-lapse imaging for 24 h in a 5% CO_2_ incubator. The cells were prestained with bisbenzimide H33342 fluorochrome trihydrochloride (H33342, 1 *μ*g/mL; Nacalai) and CytoPainter Lysosomal Staining Kit (Abcam, Tokyo, Japan) for 2 h. Then, the cells were washed once and exposed to MWNT-7 (10 *μ*g/mL). MWNT-7 uptake was snapshot-imaged at 2, 6, and 24 h, and time-lapse imaging was performed at 10 min intervals for 24 h by using differential interference contrast (DIC) and fluorescence imaging by fluorescence microscopy with cell culture equipment (AxioObserver Z1, Zeiss, Jena, Germany) using a 40x objective.

### 2.6. Assessment of MWNT-7 Uptake by Flow Cytometry

Cells were cultured on a 12-well plate for 24 h in a 5% CO_2_ incubator. Endocytosis inhibitors were pretreated 15 min before CNT exposure. Then, the cells were exposed to MWNT-7 (10 *μ*g/mL) and incubated for 2 h. The evaluation of cellular uptake for MWNT-7 followed the method reported that we reported previously [28]. In brief, the cells treated with or without MWNT-7 were washed twice and trypsinized. The cells suspended in PBS containing 10% FBS were filtered through a nylon mesh. Then, the cells were assayed for side scatter (SSC) by light scattering analysis using a flow cytometer (FCM; FACSCalibur, Becton Dickinson, San Jose, CA, USA). The SSC ratio was calculated by dividing the MWNT-7 value with the control value.

### 2.7. Statistical Analysis

Data are presented as the mean ± standard deviation (SD). Statistical significance was determined by analysis of variance (ANOVA) followed by Student's *t*-test, and *P* < 0.05 was considered to be significant.

## 3. Results and Discussion

### 3.1. Cellular Uptake by HBECs and HMCs

**Figure 1:**
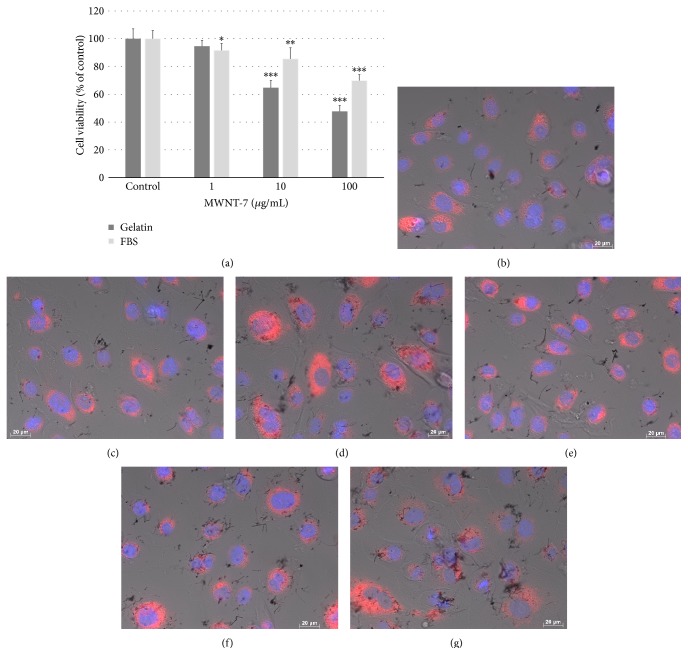
HBECs were exposed to MWNT-7. (a) Viability of HBECs exposed to various concentrations of MWNT-7 in 0.1% gelatin or 2% FBS for 24 h. HMCs were compared with HBECs exposed to MWNT-7 in each type of dispersant and to the control. Mean ± SD. *n* = 6, ^∗^ *P* < 0.05, ^∗∗^ *P* < 0.01, and ^∗∗∗^ *P* < 0.001. Image of HBECs exposed to 10 *μ*g/mL MWNT-7 in 0.1% gelatin at 2 h (b), 6 h (c), and 24 h (d) and in 2% FBS at 2 h (e), 6 h (f), and 24 h (g). DIC and fluorescence images were merged. Nuclei were stained blue with H33342 and lysosomes were stained red with CytoPainter.

First, we determined whether CNTs could be internalized in normal human bronchial epithelial and mesothelial cells, for which potential carcinogenicity of CNTs is of concern. Although we had already shown that human mesothelioma cells and commercialized normal HBECs from Cell Application internalized CNTs [28, 32, 36], Nagai et al. reported that normal human primary cultured mesothelium cells did not internalize CNTs [16]. We used HBECs purchased from a different company from that used in the previous paper to evaluate the influence of supplier on cellular uptake of CNTs. Moreover, we compared FBS as a dispersant for CNTs with gelatin in HBECs because the dispersion of CNTs by 2% FBS was recommended by the ENPRA [37]. The viability of HBECs from Lonza at 10 *μ*g/mL MWNT-7 for 24 h was approximately 85.5% in FBS and 64.7% in gelatin, and the cell viability decreased at higher concentrations (100 *μ*g/mL) in both dispersants in a concentration-dependent manner (69.9% versus 47.7%; Figure 1(a)). We observed cells dyed with fluorescence to determine whether CNTs were internalized in the cells. The visualized cells began to internalize MWNT-7 dispersed in not only gelatin but also FBS within 2 h in some cells, and uptake of MWNT-7 was observed in most cells within 6 h (Figures 1(b), 1(c), 1(e), and 1(f)). At 24 h, MWNT-7 appeared to accumulate in lysosomes (Figures 1(d) and 1(g)). Because the purpose of this paper was to elucidate the mechanisms underlying the endocytosis of CNTs, the CNTs used in subsequent experiments were dispersed with gelatin to prevent the influence of unknown factors.

**Figure 2:**
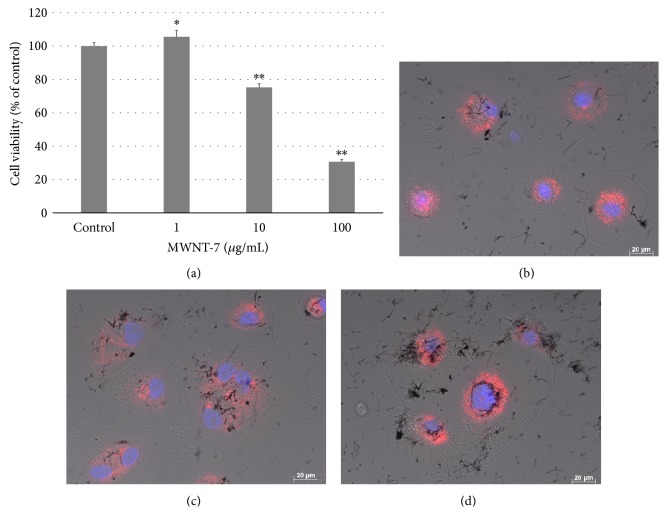
HMCs were exposed to MWNT-7. (a) Viability of HMCs exposed to variety concentration of MWNT-7 in 0.1% gelatin for 24 h. *P* values were compared to HMCs exposed to MWNT-7 in dispersant control. Mean ± S.D. *n* = 6, ^∗^ *P* < 0.05, ^∗∗^ *P* < 0.001. Image of HMCs exposed to 10 *μ*g/mL MWNT-7 in 0.1% gelatin at 2 h (b), 6 h (c), and 24 h (d). DIC and fluorescence images were merged. Nuclei were stained blue with H33342 and lysosome were stained red with CytoPainter.

Although the viability of HMCs exposed to MWNT-7 dispersed in gelatin decreased in a concentration-dependent manner (Figure 2(a)) the cell viability was still higher than that of HBECs. HMCs also began to internalize MWNT-7 within 2 h, and the internalization of MWNT-7 increased over time (Figures 2(b)–2(d)). We previously reported that BEAS-2B cells derived from human bronchial epithelium and MESO-1 cells derived from human malignant mesothelioma showed cytotoxicity arising from lysosomal injury [35]. Human normal bronchial epithelial and mesothelial cells also showed MWNT-7 internalization and cytotoxicity dependent on MWNT-7, which accumulated in the lysosome in excessive concentrations. Although Nagai et al. found that human primary mesothelium cells exposed to MWCNTs did not internalize the MWCNTs based on the SSC ratio, transmission electron microscopy, confocal microscopy, and time-lapse microscopy, they evaluated the results at 3 h after exposure to the materials [16]. We speculate that these results were obtained because it is difficult for CNTs to sink in the solution owing to their very light weight. In fact, our results showed that uptake of MWNT-7 observed by DIC increased over time, and only a few cells internalized MWNT-7 in 2 h. It has also been reported that the quantity of CNTs that undergo cellular uptake increases until approximately 12 h [38]. Moreover, although previous studies have evaluated the uptake of CNTs in comparison with asbestos, such a comparison under the same conditions is not effective because cellular uptake of different materials depends on their physicochemical properties. Another study showed that MWCNT exerted adverse effects without CNT uptake in a human mesothelial cell line (Met-5A) [39]. However, although they showed transmission electron microscopy (TEM) images of A549 alveolar epithelial cell lines to conclude that the cells do not internalize CNTs, no TEM images for Met-5A were presented, and optical microscope images of low magnification (×20) were only shown. Lindberg et al. reported genotoxicity of MWCNTs based on TEM images showing that the Met-5A cells internalize CNTs [40]. Moreover, our time-lapse data clearly and directly indicate that HBECs and HMCs endocytose MWCNTs actively (Movies S1 and S2) (see Supplementary Material available online at http://dx.doi.org/10.1155/2015/793186). We also have already reported that the BEAS-2B cell line derived from human bronchial cells and MESO-1 cells derived from human mesothelioma cells internalized some MWCNTs [28, 36]. Therefore, we used inhibitors of endocytosis, to clarify the internalization mechanism of CNT further using BEAS-2B cells and MESO-1 cells rather than HBECs and HMCs, respectively.

We investigated the mechanism of CNT uptake using inhibitors for three endocytosis pathways (clathrin-mediated, caveolae-mediated, and macropinocytosis), with the SSC ratio as an index. We have already shown that SSC ratio increases concentration dependently over time in cells that only internalized CNTs [28]. The SSC ratios of the control, which was not pretreated by inhibitors in BEAS-2B and MESO-1 cells, were 1.355–1.426 and 1.137–1.258 in 2 h, respectively. It was observed that the SD of the SSC ratios tended to increase with cell passage number, likely because we analyzed under sixteen passages for both cell lines. Therefore, few statistically significant differences were noted when we assayed the SSC ratios of nystatin as a caveolae-mediated endocytosis inhibitor and 5-(N-ethyl-N-isopropyl)amiloride as a macropinocytosis inhibitor.

**Figure 3:**
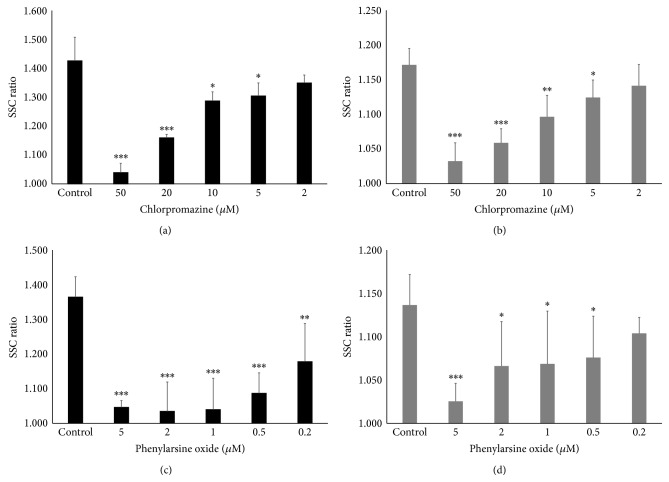
Effect of clathrin-mediated endocytosis inhibitors on cellular uptake of MWNT-7. The SSC ratios of (a) BEAS-2B cells and (b) MESO-1 cells pretreated with various concentrations of chlorpromazine are shown. The SSC ratios of (c) BEAS-2B cells and (d) MESO-1 cells pretreated with various concentrations of phenylarsine oxide are shown. The cells were compared with control cells pretreated with inhibitor solvent. Mean ± SD. *n* = 4 or 6, ^∗^ *P* < 0.05, ^∗∗^ *P* < 0.01, and ^∗∗∗^ *P* < 0.001.

Two clathrin-mediated endocytosis inhibitors suppressed the ratio in a concentration-dependent manner in both cell lines (Figures 3(a)–3(d)). In BEAS-2B cells, the maximum concentration of chlorpromazine (50 *μ*M) decreased the SSC ratio to 1.039, whereas the SSC ratio with 2 *μ*M phenylarsine oxide was 1.040. In MESO-1 cells, the lowest SSC ratios were 1.032 and 1.025 with treatment with 50 *μ*M chlorpromazine and 5 *μ*M phenylarsine oxide, respectively. Because the baseline SSC ratio for which the cells were not exposed to CNTs was 1.000, clathrin-mediated endocytosis seems to be the main mechanism for cellular uptake.

**Figure 4:**
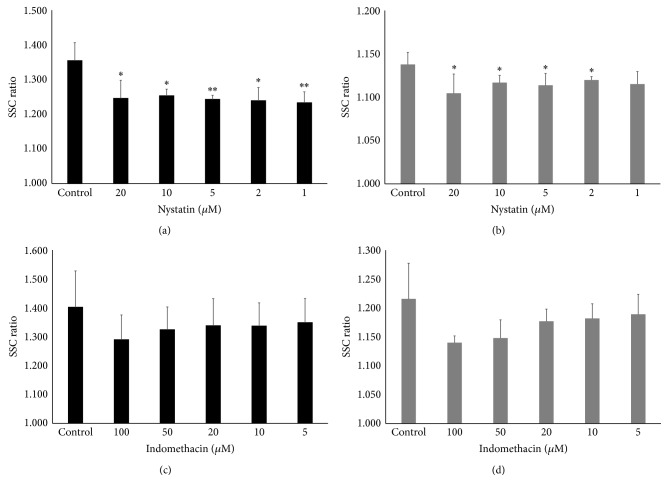
Effect of caveolae-mediated endocytosis inhibitors on cellular uptake of MWNT-7. The SSC ratios of (a) BEAS-2B cells and (b) MESO-1 cells pretreated with various concentrations of nystatin are shown. The SSC ratios of (c) BEAS-2B cells and (d) MESO-1 cells pretreated with the various concentrations of indomethacin are shown. The cells were compared with control cells pretreated with inhibitor solvent. Mean ± S.D. *n* = 4, ^∗^ *P* < 0.05, ^∗∗^ *P* < 0.01.

The results for caveolae-mediated endocytosis inhibitors were complicated. Nystatin decreased the SSC ratio in both cells significantly except at 1 *μ*M in MESO-1 cells (Figures 4(a) and 4(b)). In detail, MESO-1 cells displayed a tendency for concentration dependency, whereas the inhibition did not depend on the dose in BEAS-2B cells. In contrast, although indomethacin tended to show concentration-dependent inhibition in both cell lines, there was no statistically significant difference (Figures 4(c) and 4(d)). The difference in the results may be caused by the inhibition mechanism. Nystatin disrupts caveolar function and binds to sterol in the plasma membrane [41–43]; indomethacin blocks the internalization of caveolae and the return of plasmalemmal vesicles [44, 45]. However, we considered that caveolae-mediated endocytosis pathway may partially contribute to the internalization of CNTs for the following reasons: (1) the inhibition rate of nystatin, which was not concentration-dependent, was 30.7% and was the same as the inhibition rate (27.9%) with the highest concentration of indomethacin (100 *μ*M) in BEAS-2B cells. (2) In MESO-1 cells, both inhibitors showed a tendency for concentration-dependence, and the inhibition rate provided by indomethacin, which inhibits the essential parts of the endocytosis pathway, was higher than that by nystatin (35.2% and 23.9%, resp.). The inhibition of statin binding to the sterol may have been responsible for difference among cell types.

**Figure 5:**
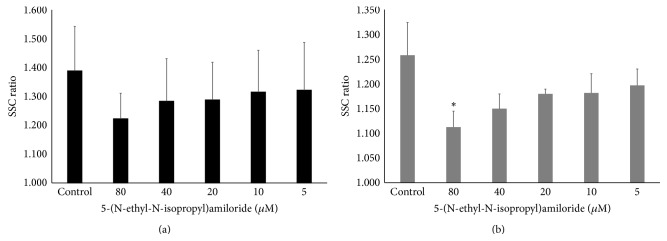
Effect of a macropinocytosis inhibitor on cellular uptake of MWNT-7 cells. The SSC ratios of (a) BEAS-2B cells and (b) MESO-1 cells pretreated with various concentrations of 5-(N-ethyl-N-isopropyl)amiloride are shown. The cells were compared with control cells pretreated with inhibitor solvent. Mean ± SD. *n* = 3, ^∗^ *P* < 0.05.

5-(N-Ethyl-N-isopropyl)amiloride, which inhibits the macropinocytosis pathway, seems to suppress CNT uptake in a concentration-dependent manner, although the difference did not reach significance except at 80 *μ*M in MESO-1 cells (Figures 5(a) and 5(b)). The inhibition rates of BEAS-2B cells and MESO-1 cells were comparable at 42.7% and 56.6% at the highest concentration (80 *μ*M). The role of macropinocytosis in CNT uptake has not been extensively studied. Hirano et al. demonstrated that macrophage receptor with collagenous structure- (MARCO-) transfected CHO-K1 cells takes up MWCNTs via membrane ruffling in a process similar to macropinocytosis [46]. They also reported that MARCO was absorbed in MWCNTs to which macrophages were exposed [47]. However, it was not clear whether macropinocytosis for CNTs occurs in nonphagocytic cells. Our results indicate that macropinocytosis plays an important role in CNTs uptake.

The latest information for cellular uptake of nanomaterials has been reviewed and suggests that three different mechanisms of endocytosis exist including clathrin- and caveolae-independent endocytosis, and also endocytosis depends on particle physical-chemical properties, experimental conditions, and cell type in nonphagocytic cells [48]. We consider that CNT uptake is also subject to the same influences in an interdependent manner because the total inhibition rate when independently inhibited pathways were considered together easily exceeded 100%, which means that other pathways function in a compensatory manner, even when one pathway is inhibited. Moreover, other pathways may exist because there are some reports indicating several types of clathrin- and caveolae-independent endocytosis, and the endocytic mechanism is especially unexplained in the nonphagocytic cells [49–51]. In fact, it was not possible to clarify the mechanism underlying the observed suppression of CNT uptake in BEAS-2B cells cultured in FBS-free medium [32]. That study also indicated that the degree of aggregation is an important factor but we could not clarify this issue. We measured the SSC ratio in the comparatively early stage of 2 h after CNT exposure because high concentrations of the inhibitors showed cytotoxicity. Within 2 h, a small fibrous agglomerate containing some MWCNTs was seen at the bottom of the dish. Although it seems likely that our inhibitor results reflect actual endocytosis, it is unclear whether the nonagglomerated MWCNTs observed after 2 h at the bottom in Movie S1 and Movie S2 show the same result. However, there appeared to be a common cellular uptake pattern for the MWCNTs.

In conclusion, we found that human normal bronchial epithelial cells and mesothelium cells endocytosed MWCNTs. The mechanism of endocytosis seemed to be not only one but a combination of three pathways: clathrin-mediated endocytosis, caveolae-mediated endocytosis, and macropinocytosis. Although clathrin-mediated endocytosis played the most important role, other pathways may be involved to varying degrees. The cellular uptake of MWCNTs is essential for MWCNT toxicity in the context of genotoxicity. It may thus be necessary to prepare materials that are not endocytosed to develop the nanomaterials having not only useful but also hazardous properties, as we alluded to in a previous study [28]. We have already reported that both BEAS-2B and MESO-1 cells did not endocytose MWCNTs dispersed in carboxymethyl cellulose. Therefore, this and previous studies suggest that biocompatible nanomaterials can be developed.

## Supplementary Material

Movie S1: Time-lapse imaging of HBECs that were exposed to 10 μg/mL MWNT-7 in 0.1% gelatin at 10 min for 24 h using fluorescence microscopy. Lysosomes were prestained red with CytoPainter.
Movie S2: Time-lapse imaging of HMCs which were exposed to 10 μg/mL MWNT-7 in 0.1% gelatin at 10 min for 24 h using fluorescence microscopy. Lysosome were prestained red with CytoPainter.
